# Practical Notes and Formulæ

**Published:** 1888-09

**Authors:** 


### PRACTICAL NOTES AND FORMULÆ.

**For Distressing Cough in Phthisis.**—By R. Kindig, M. D., in *Illustrated Medical News.*

℞. Chlorodyne.............................ʒ iss,

Syr. scillæ.................................℥ ss,

Spt. ammonii aromat.........................ʒ iij.

Aquæ destillatæ.............................ʒ ij.

M. Sig.—Desertspoonful three times daily.

**Dyspepsia, with Chronic Constipation and Sour Stomach.**—*Ib.*

℞. Fl. ext. berberis aquifolium.............℥ ss,

Fl. ext. cascara sagrada....................ʒ ij,

Tinct. nux vomica...........................gtts. x.

Dilute Hydrocyanic acid.....................ʒ j,

Ext. malt, q. s. ad.........................℥ j.

M. Sig.—A teaspoonful half an hour after each meal.

**Ring Worm.**—By Dr. H. H. Dorr, Caldwell, Texas.—*Ib.*

℞. Ammoniated mercury.........................

Flowers sulphur.............................aaʒ j,

Vaseline....................................℥ j.

M. Sig.—Apply to parts affected once or twice a day. I have used this with almost uniform success.

**Simple Treatment of Itch.**—*Revue Ther.*

℞. Animal fat.............................gram. cxxv,
Benzine.....................................gram. xxx.

Three or four frictions with the above ointment, followed by an alkaline bath.

**Treatment of Meningetis in Children.**—H. Pierron, *Revue de Ther.* 1. Keep the bowels open by calomel taken in the morning.

2. Apply fly-blisters to the head.

3. Rub the thighs and groins with Neapolitan ointment three or four times a day.

4. Give of the following prescription a teaspoonful every half hour to a child of two years:

℞. Bromide of potass.......................gram. 3,
Iodide of potass............................gram. 0.60,
Tincture of musk............................gtts. x,
Syrup of quinine............................gram. xxx,
Aromatic water..............................gram. cxx.

Apply leeches to the mastoid process.

**Hemorrhages of the Lungs and all Dangerous Internal Hemorrhages.**—*Medical Age.*

℞. Saturated tincture secale cornutum............
Saturated tincture cinnamonum cort............
Saturated tincture ol. erigeron canadensis....aa℥ j.

M. Sig.—Thirty to sixty drops on sugar, or in mucilage, and repeat as often as required until the hemorrhage is controlled, when smaller doses should be given on extended time. For local hemorrhage of any part of the body, where this preparation can be applied, it will be found of great efficacy as a safe styptic, especially in epistaxis.

**Incontinence of Urine in Children.**—*Medical Age.*

℞. Sat. tin. gnaphalium plantaginifolia (Mouse Ear)..℥ ij,

Sig.—Thirty to sixty drops every night, at bed-time. Where a tincture cannot be obtained, an infusion may be substituted.

**Lugol's Solution.**—(Liquor Iodi Compositus) *Ib.*

℞. Iodi....................................parts v,
Potassi iodidi.............................. " x,
Aquæ destillatæ............................ " lxxxv.

M. S.—Dose 1 to 10 *m.*, well diluted.

**H. C. Woods on Colds.**—For general "*cold*," a free jaborandi sweat and quinine in full does. These may be assisted by mercurial or other purgatives. For coryza, bismuth and cocaine injections. In bronchitis expectorants: chloroform is one of the most valuable to quiet cough, as whiskey, paregoric, glycerine, each ʒij, chloroform, *m* xxx. Teaspoonful doses, shaking. Instead of the depressing expectorants, he uses:

℞. Potass. citrat...............................℥ j,

Succi limonis...............................℥ iss,

Syr. ipecac.................................℥ ss,

Tr. opii., camph.............................ʒ iij,

Syrupi qs. ad...............................℥ iij.

Desertspoonful or less every two hours. This failing he gives chloride of ammonium 7½ grain doses every two hours, disguised by an equal quantity of ext. glycyrrhizæ, or in capsules followed by water.—*Therapeutic Gazette.*

**Typhoid Fever.**—The interesting observation is made by Dr. Underwood, Customs Medical Officer at Kiukiang, China, that the comparative immunity of the Chinese in that region from typhoid fever, notwithstanding most of the factors favoring it are present in abundance, may be attributed to the fact that "cold," unboiled water is rarely or never used when tea can be had.—*New York Medical Record.*

